# Analysis of Interictal Epileptiform Discharges in Mesial Temporal Lobe Epilepsy Using Quantitative EEG and Neuroimaging

**DOI:** 10.3389/fneur.2020.569943

**Published:** 2020-11-26

**Authors:** Elaine Keiko Fujisao, Karen Fernanda Alves, Thais O. P. Rezende, Luiz Eduardo Betting

**Affiliations:** Departamento de Neurologia, Psiquiatria e Psicologia, Faculdade de Medicina de Botucatu, UNESP - Universidade Estadual Paulista, Botucatu, Brazil

**Keywords:** mesial temporal lobe epilepsy, magnetic ressonance imaging, eletroencepalogram, hippocampal atrophy, quantitative EEG, quantitative MR analysis

## Abstract

**Objective:** Investigate areas of correlation between gray matter volumes by MRI and interictal EEG source maps in subtypes of mesial temporal lobe epilepsy (MTLE).

**Method:** 71 patients and 36 controls underwent 3T MRI and and routine EEG was performed. Voxel-based morphometry (VBM) was used for gray matter analysis and analysis of interictal discharge sources for quantitative EEG. Voxel-wise correlation analysis was conducted between the gray matter and EEG source maps in MTLE subtypes.

**Results:** The claustrum was the main structure involved in the individual source analysis. Twelve patients had bilateral HA, VBM showed bilateral hippocampal. Twenty-one patients had right HA, VBM showed right hippocampal and thalamic atrophy and negatively correlated involving the right inferior frontal gyrus and insula. Twenty-two patients had left HA, VBM showed left hippocampal atrophy and negatively correlated involving the left temporal lobe and insula. Sixteen patients had MTLE without HA, VBM showed middle cingulate gyrus atrophy and were negatively correlated involving extra-temporal regions, the main one located in postcentral gyrus.

**Conclusions:** Negative correlations between gray matter volumes and EEG source imaging. Neuroanatomical generators of interictal discharges are heterogeneous and vary according to MTLE subtype.

**Significance:** These findings suggest different pathophysiological mechanisms among patients with different subtypes of MTLE.

## Introduction

Interictal spikes in the EEG of patients with mesial temporal lobe epilepsy (MTLE) critically contribute to the diagnosis and lateralization of the epileptogenic zone (1, 2). Seizures can originate from one or more anatomical structures of the temporal lobe and spread inward (temporal) or outward (extratemporal) through connected neural networks (3).

Anatomical generators of epileptiform discharges in MTLE have not yet been accurately identified. The maximal negativity of epileptiform activity is in middle and posterior temporal electrodes suggesting a possible onset in the temporal neocortex (4). Intracranial recordings and magnetoencephalograpy (MEG) also support a temporal origin and indicate that conventional noninvasive EEG and MEG cannot identify true mesial temporal spikes (5).

Using quantitative EEG one investigation demonstrated that 62% of MTLE patients had dipole source localization in the epileptogenic temporal lobe (6). Two main types of dipole orientation were described, one with vertical orientation corresponding to inferior or basal temporal spikes and the second with horizontal orientation corresponding to lateral temporal spikes. The second showed widespread cortical thinning when compared to the fisrt one: in the left hemisphere involving cingulate, supramarginal, occipitotemporal an parahippocampal gyri, precuneus and parietal lobule; in the right hemisphere, frontomedial, central and basal gyri and precuneus was envolved. These findings suggest that a large cortical network in this group is affected and showed that the distribution of interictal spikes was associated with widespread cortical thinning beyond the mesiotemporal region (7, 8).

MTLE pathophysiology is not limited to the amygdalo-hippocampal system. Quantitative MRI revealed an extensive network of abnormal neural structure in these patients (9). In-depth investigation showed that in unilateral MTLE the ipsilateral hippocampus is markedly abnormal, and extrahippocampal cortical regions (including the precentral and paracentral gyri) show decreased thickness (10).

The relationship between structural and neurophysiological observations is not completely understood. The objective of the present study was to investigate correlations between gray matter volumes and interictal EEG source maps in subtypes of MTLE. It was hypothesized that this correlation may be present and would change according to subtypes of MTLE. This observation could help to map the epileptogenic circuitry involved in the pathophysiology of MTLE and improve understanding of the mechanisms of interictal epileptiform discharges observed in these patients.

## Methods

### Subjects

Seventy-one patients with MTLE (51 women, mean age 46.2 ± 12.6 years) and 36 control subjects (18 women, mean age 32 ± 7 years) with no history of epilepsy or neurological diseases were investigated. Patients were selected at the Outpatient Epilepsy Clinic of the Hospital das Clínicas of Botucatu Medical School. The diagnosis of MTLE was assigned according to clinical and electroencephalographic criteria (1). All patients had at least one typical EEG with temporal epileptiform discharges. Patients with extra-temporal pathology, double pathology or non-epileptic seizures were excluded. Controls were randomly recruited from the local community. All participants signed an informed consent form approved by the local Ethics Committee.

### Electroencephalography Acquisition

Patients were submitted to EEG with a 32-channel Nihon-Kohden (Tokyo, Japan) system. EEG acquisition used the international 10-20 electrode placement system and 1,000 Hz sample rate, with additional Silverman T1 and T2 electrodes. In all 23 electrodes impedance was kept below 10 kΩ and duration of EEG was approximately 20 min including photostimulation and hyperventilation.

### Analysis of Interictal Discharges

**Figure 1:**
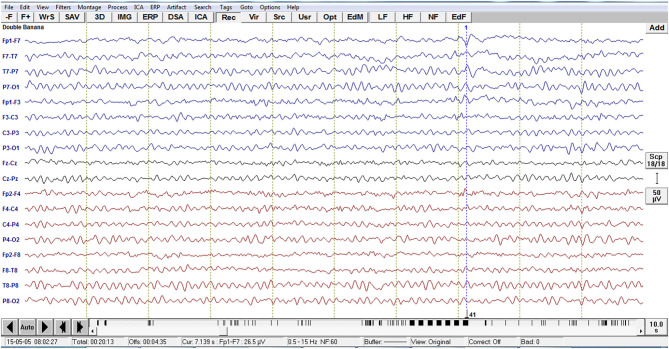
Example of EEG imported to the software BESA with the left temporal epileptiform activity with phase reversal with equipotentiality F7 -T1. For each individual, all epileptiform discharges were manually selected.

**Figure 2:**
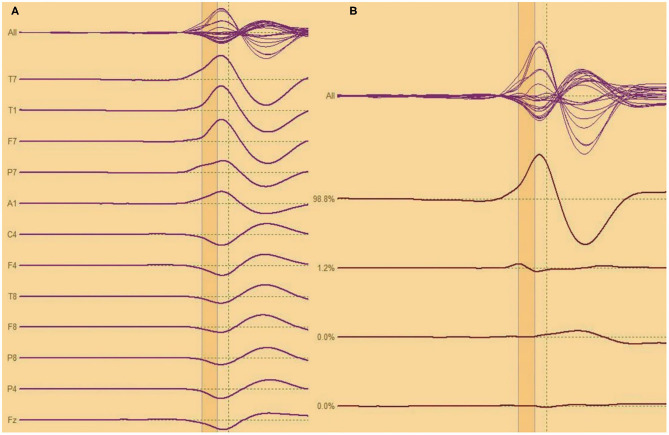
**(A,B)** Analysis of interictal discharge for each individual all epileptiform discharges were averaged. A 10 ms segment in the ascending portion of the averaged epileptiform activity was then selected.

**Figure 3:**
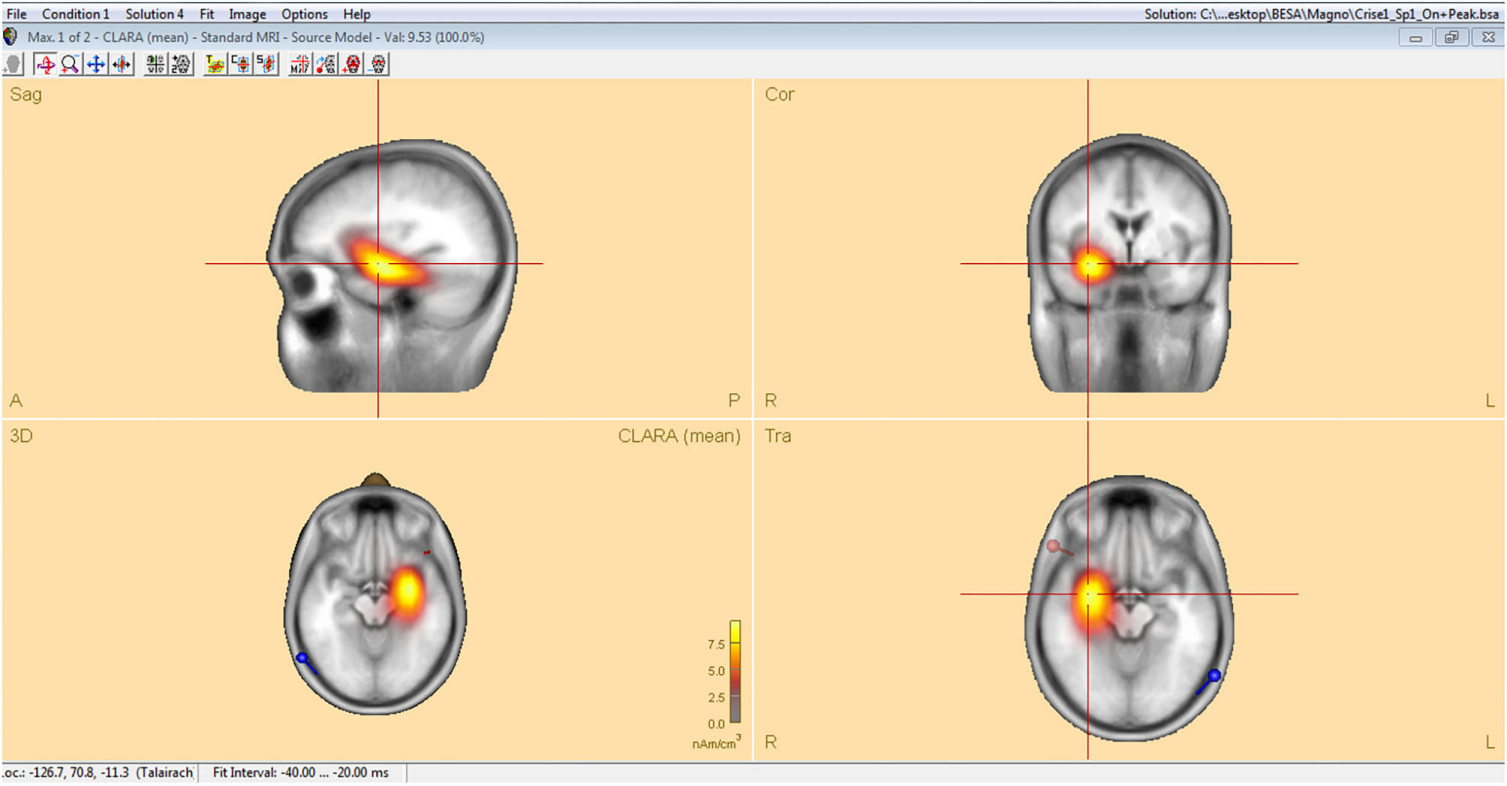
Classical LORETA analysis recursively applied (CLARA) algorithm included in the BESA package used to generate epileptiform discharges source maps from a realistic approximation model for adult patients, according to Tailarach coordinates extracted as three- dimensional high-resolution files (1 mm) in ANALYZE format.

Analysis of interictal discharge sources was performed using BESA Research 6.0 software (BESA GmbH, Gräfelfing, Germany). EEG was imported to the software and for each individual all epileptiform discharges were manually selected and averaged (Figure 1). A 10 ms segment in the ascending portion of the averaged epileptiform activity was then selected, guided by principal component analysis, and was maintained with an average of 97.7% for left discharges and 97.3% for right discharges, which means that the analysis represents the majority of the spikes in both sides (Figure 2). For patients with more than one EEG or bilateral discharges the exams were individually analyzed and the discharges were grouped by side and also separately analyzed. Classical LORETA Analysis Recursively Applied (CLARA) algorithm included in the BESA package was used to generate epileptiform discharges source maps using a realistic approximation model for adult patients. Peak locations of the discharges maps were extracted according to Tailarach coordinates (11). Source images were exported as three-dimensional high resolution files (1 mm) in ANALYZE format (Figure 3). These images were used to calculate mean and overlap images for the MTLE subgroups (12).

### Magnetic Resonance Imaging Acquisition

All subjects underwent MRI. Images were acquired using a 3T scanner (Siemens, Verio, Erlangen, Germany) with a 12-channel head coil. The acquisition protocol included magnetization-prepared rapid gradient-echo (MPRAGE) with 0.5 × 0.5 mm voxels, acquired with 1 mm thickness in the sagittal plane; excitation angle 9°; TR (repeat time) 2,300 ms; TE (excitation time) 2.47 ms; TI (inversion time) 1,100 ms; matrix 256 × 256 and field of view (FOV) 256 mm; Axial FLAIR (Fluid Attenuated Inversion Recovery) 4.0 mm thick; excitation angle 150°; TR 9,000 ms; TE 80 ms; TI 2,500 ms; 208 × 256 matrix and FOV 220 mm; Coronal T2 perpendicular to the long axis of the hippocampus with 2.2 mm of thickness; excitation angle 120°; TR 4,000 ms; TE 135 ms; matrix 230 × 256 and FOV 180 mm; coronal T1 STIR (Short TI Inversion Recovery) perpendicular to the long axis of the hippocampus with 2.2 mm thickness; excitation angle 150°; TR 2,100 ms; TE 9.5 ms; TI 499 ms; matrix 230 ×256 and FOV 180 mm; coronal T2 STIR perpendicular to the long axis of the hippocampus with 2.2 mm of thickness; excitation angle 150°; TR 4,300 ms; TE 74 ms; matrix 128 × 128 and FOV 180 mm. Quantitative structural analysis was performed using the T1 volumetric sequence. Qualitative assessment of hippocampus and evaluation of other cerebral lesions was performed with the remainder sequences.

### MRI Quantitative Analysis

Automatic volumetry was conducted using standard routines within FreeSurfer software (version 6.0, https://surfer.nmr.mgh.harvard.edu/) (13, 14). This program automatically normalizes intensity, corrects for movement artifacts, removes extra-cerebral tissues, registers the images into Talairach space, and segments subcortical structures including hippocampus (15). For each subject, hippocampal volume was normalized to total intracranial volume (16). Across groups, hippocampal volumes were normalized to those of controls (yielding a z score). Asymmetry index was calculated as 2 × (right hippocampal volume – left hippocampal volume) / (right hippocampal volume + left hippocampal volume). HA was defined as when the standardized volumes were at least 2 standard deviations (SDs) less than the mean, or when the asymmetry index was at least 2 SDs from the mean in either direction.

Based on the volumetry findings, patients were divided into four groups: bilateral HA (BHA), right HA (RHA), left HA (LHA) and without HA (normal). Voxel-based morphometry (VBM) was performed using the SPM12 program (http://www.fil.ion.ucl.ac.uk) running on MATLAB® R2012b platform. This program performs a unified segmentation (17). Initially the gray matter of all subjects was automatically segmented. Then, using the Diffeomorphic Anatomical Registration Through Exponentiated Lie Algebra (DARTEL) algorithm, a template was created based on all individuals included in the study. This template was registered into standard ICBM-152 (International Consortium for Brain Mapping) space. The final step was to register the segmented images with the template. Structural images were also modulated with the objective of preserving gray matter and minimizing distortions due to normalization. A Gaussian filter (8 mm Full Width at Half Maximum) was applied to all segmented images and EEG maps in order to reduce variation and normally distribute the intensity of the voxels.

### Correlation and Statistical Analysis

Voxel wise comparisons were made by full factorial design, looking for areas of increased or decreased gray matter. The four patient groups were compared to controls. A *p* value < 0.05, corrected for multiple comparisons (Bonferroni correction), was the threshold for statistical significance. Age, sex, and total intracranial volume were introduced as covariates.

For the four groups, voxel by voxel correlation analysis was also performed between the interictal discharge source maps and gray matter volume maps using SPM5 and BPM program (18). Regions of interest analysis were also performed for VBM and for correlation analysis, using the same parameters. Three analyzes were performed, the first global analysis, the second involving the temporal lobes and the third including only the hippocampi. The regions were selected according to the AAL (Automatic Anatomic Labeling) atlas (19–21).

## Results

### Clinical Features

Of the 71 MTLE patients studied, average age of seizure onset was 18.4 ± 15.7 years and frequency of seizures was 7.1 ± 13 seizures per month. Twelve patients had bilateral HA (8 females, 41.1 ± 14.6 years) with standardized mean volumes of −5.6 ± 1.9 SDs and −4.2 ± 1.4 for the left and right hippocampus, respectively. Twenty-one patients had right HA (15 women, mean age 45 ± 12 years) with mean hippocampal volumes of 1.0 ± 1.4 SDs (left) and −3.8 ± 1.6 (right). Twenty-two patients had left HA (15 women, mean age 46.3 ± 10.9 years), mean normalized volumes were −3.5 ± 1.9 SDs for the left and 1.3 ± 2.3 for the right hippocampus. Sixteen patients had normal MRI without HA (12 females, 51.2 ± 13.3 years), and standardized mean hippocampal volumes of 0.7 ± 1.9 SDs and 0.8 ± 1.8 for the left and right hippocampus, respectively. Additional characteristics can be found in Table 1 and Appendix.

**Table 1:** Clinical, electroencephalographic, and neuroimaging features of the four groups of patients with mesial temporal lobe epilepsy (bilateral, right and left hippocampal atrophy, and without hippocampal atrophy).

	**Bilateral**	**Right**	**Left**	**Normal**
n (%)	12 (16.9%)	21 (29.6%)	22 (30.9%)	16 (22.6%)
Refractory (%)	11 (91.7%)	20 (95.2%)	21 (95.4%)	11 (68.75%)
First seizure	10.3 ± 9.6 (1–24)	12.0 ± 10.2 (1–39)	19.2 ± 16.5 (1–55)	31.4 ± 16.5 (10–75)
Duration	31.0 ± 13.8 (10–55)	34.1 ± 15.8 (10–60)	32.6 ± 16.7 (4–55)	17.3 ± 15.5 (1–48)
Frequency	4.25 ± 5.8 (0–20)	10.9 ± 20.2 (0–90)	7.7 ± 10.0 (0–36)	3.1 ± 5.6 (0–20)
AEDs	1.9 ± 0.5 (1–3)	2.1 ± 0.6 (1–3)	2.4 ± 1 (1–5)	1.2 ± 0.4 (1–2)
Age	41.2 ± 14.6 (18–61)	45.0 ± 12.0 (20–60)	46.3 ± 10.9 (30–61)	51.2 ± 13.3 (31–80)
Gender	8 women	15 women	15 women	12 women
Vol RH	2522 ± 421 (1949–3504)	2603 ± 379 (1888–3526)	3510 ± 338 (3000–4260)	3508 ± 522 (2671–4245)
z RH	−4.2 ± 1.4 (−6.9 to−2.2)	−3.8 ± 1.6 (−6.9–0.7)	1.3 ± 2.3 (−1.4–7.1)	0.8 ± 1.8 (−1.5–4.3)
Vol LH	2248.3 ± 555.7 (1828 – 3674)	3506.3 ± 181.7 (3108 – 3868)	2584.6 ± 346.2 (1961 – 3409)	3408.5 ± 467.0 (2767 – 4039)
z LH	−5.6 ± 1.9 (−8.0 to −2.0)	1.0 ± 1.4 (−1.5–4.1)	−3.5 ± 1.9 (−7.3 to −0.5)	0.7 ± 1.9 (−1.6–4.1)
z AIS	3.1 ± 3.9 (−2.0–10.3)	−9.5 ± 4.04 (−19.5 to −2.1)	8.4 ± 3.7 (1.9–15.5)	0.2 ± 0.9 (−1.6–1.6)
EEG/patient	1 ± 0 (1–1)	1.2 ± 0.4 (1–2)	1.4 ± 0.7 (1–4)	1.2 ± 0.5 (1–3)
Discharges	11.3 ± 13.4 (1–37)	36.7 ± 55.8 (5–234)	4.2 ± 3 (2–7)	12.3 ± 19 (1–55)
Right Left	37.7 ± 54.7 (2–155)	6.6 ± 18.4 (2–70)	44.9 ± 80.6 (1–359)	7.4 ± 4.8 (1–16)

*n, number of subjects in group; Refractory (%): patients remaining with at least one monthly seizure despite appropriate use of antiepileptic medications; First seizure, age of first seizure in years; Duration, time of epilepsy since the first recurrent seizure; Frequency, estimated number of monthly seizure; AEDs, number of antiepileptic medications currently in use by the patients; Age, in years; Vol RH, right (non-normalized) hippocampal volume in cubic millimeters; z RH - z, right hippocampus score; Vol LH, left hippocampal volume (non-normalized) in cubic millimeters; z RH – z, left hippocampus score; z AIS, z - asymmetry index score; EEG/patient, number of EEGs analyzed per patient; Discharges, number of epileptiform discharges analyzed by patients. Data demonstrated in the mean ± standard deviation (minimum value - maximum value)*.

### Electroencephalographic Features

Eighty-six EEGs were investigated. Eleven patients had two records, one patient had three records, and one patient four records (mean of 1.2 ± 0.5 records per patient). Thirty patients had right discharges only, 33 had left discharges only, and 8 had bilateral discharges. In total, 2,366 epileptiform discharges were observed, 1,367 left (33 ± 62 discharges per patient, range 1–359) and 959 right (25 ± 44 discharges per patient, range 1–234).

In BHA group, 264 discharges on left (37.7 ± 54.7 discharges, range 2–155) and 79 discharges on right (11.3 ± 13.4 discharges, range 1–37) were analyzed. In RHA group, 139 discharges on left (6.6 ± 18.4 discharges, range 2–70) and 771 discharges on right were analyzed (36.7 ± 55.8 discharges, range 5–234). In LHA group, 897 discharges were analyzed on left (44.9 ± 80.6 discharges, 1–359) and 21 on right (4.2 ± 3 discharges, 2–7). In normal group, 67 discharges on left (7.4 ± 4.8 discharges, 1–16) and 88 discharges on right (12.3 ± 19 discharges, 1–55) were analyzed.

**Figure 4:**
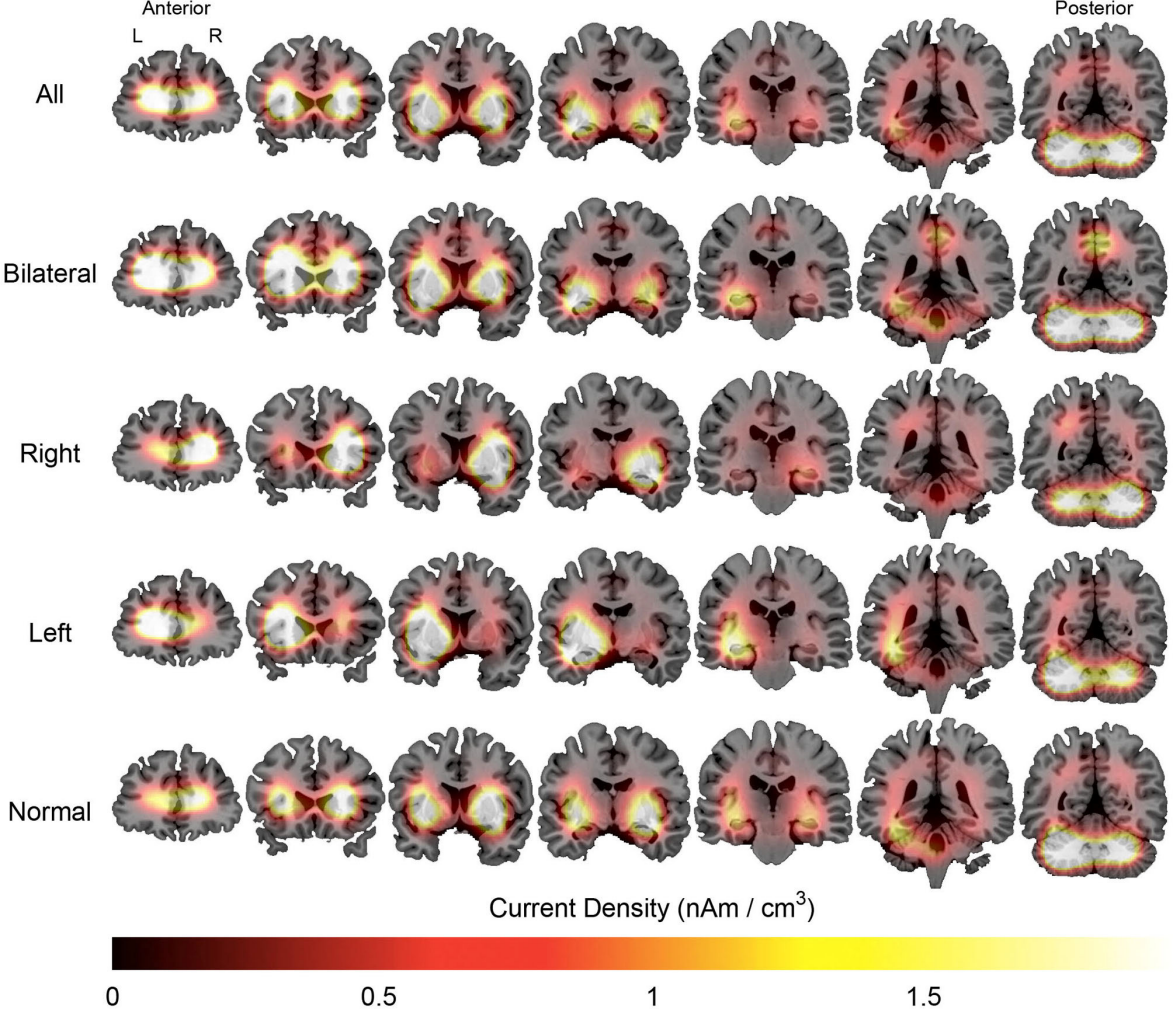
Average maps of the localization of interictal epileptiform discharges of patients with mesial temporal lobe epilepsy. The figure is color-coded according to the current density and overlaid on an MRI model (coronal section). The orientation is in neurological convention (right on right). The slices on left are anteriorly located than those to right.

**Figure 5:**
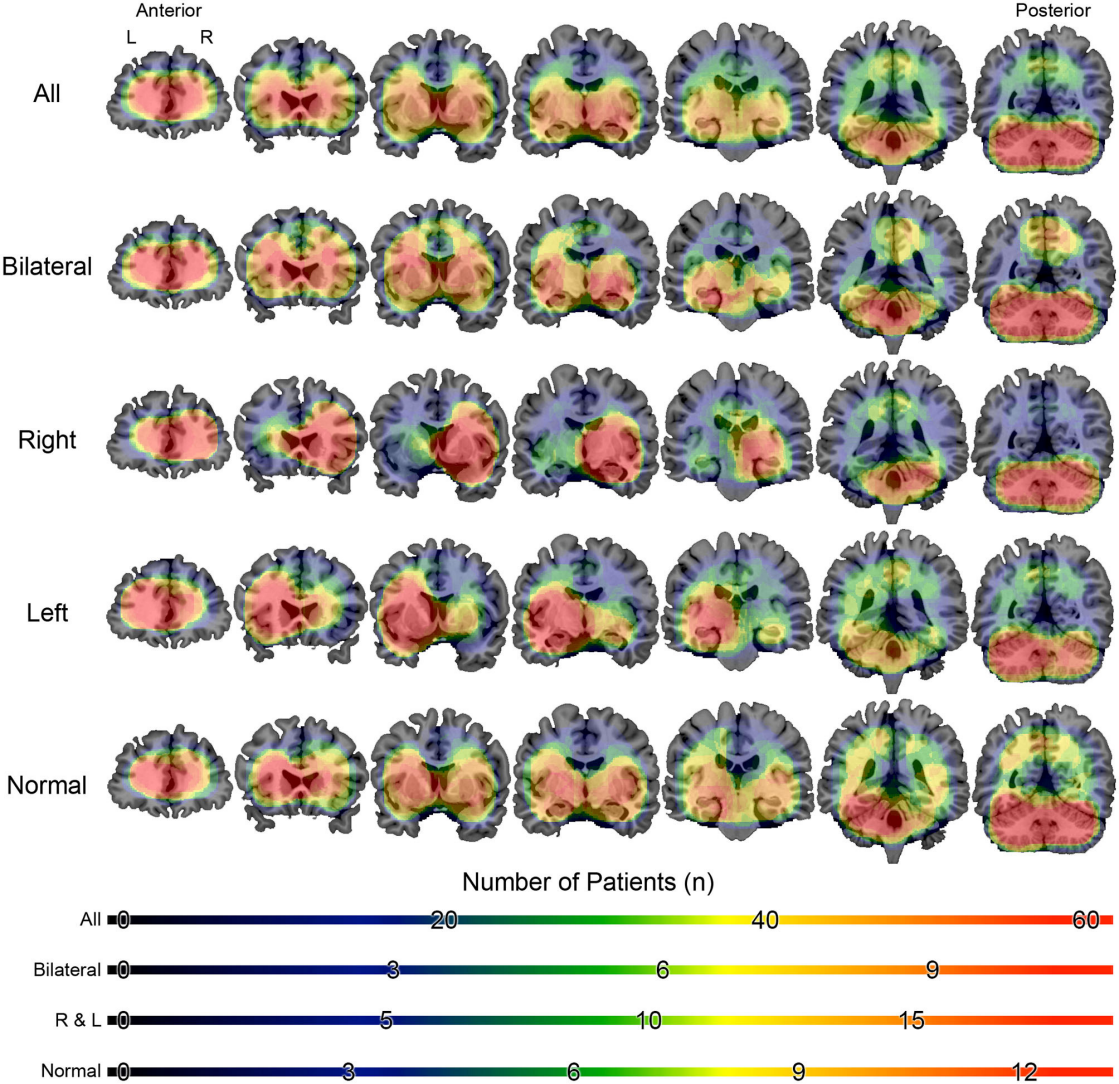
Overlap maps of the localization of interictal epileptiform discharges of patients with mesial temporal lobe epilepsy. The figure is color-coded according to the number of individuals and overlaid on a magnetic resonance model (coronal section). The figure is in the neurological orientation (right on right). The slices on left are anteriorly located than those to right.

Source analysis of the discharges showed 95 foci distributed over 16 areas. The main areas were claustrum (22%), inferior frontal region (16%), insula (12%) and superior temporal gyrus (12%). For BHA patients, the three main locations were claustrum (23%), inferior frontal gyrus (23%), and lentiform nucleus (15%). For RHA patients the three main locations were claustrum (20%), insula (20%), and inferior frontal gyrus (16%). For LHA patients the three main locations were claustrum (19%), superior temporal gyrus (19%), and inferior frontal gyrus (15%). For the normal group, the three main locations were claustrum (33%), lentiform nucleus (22%), and parahipocampal gyrus, lentiform gyrus, and precentral gyrus (11% each). Maps of discharges can be observed in Figure 4 (average) and Figure 5 (overlap of all patients).

Table 1 summarizes the clinical and electroencephalographic features, Table 2 shows the source location for all patients and Supplementary Tables shows the source location for the subgroups.

**Table 2:** Results of the source localization using the CLARA algorithm for all patients with mesial temporal lobe epilepsy.

**Anatomy**	**Right**	**Left**	**Total**
Claustrum	11	10	21
Inferior frontal	8	7	15
Insula	6	6	12
Superior temporal	5	7	12
Parahhipocampal	5	3	8
Lentiform	5	2	7
Cingulate	2	3	5
Culmen	1	2	3
Caudate	0	3	3
Middle frontal	1	1	2
Precentral	0	2	2
Middle temporal	0	1	1
Inferior temporal	0	1	1
Sub-lobar	0	1	1
Thalamus	1	0	1
White matter	1	0	1

### Voxel-Based Morphometry Analysis

The BHA group, as compared to controls, showed atrophy in seven main regions (totaling 166,853 mm^3^), the most significant region being right hippocampus. For the RHA group, only one cluster was significantly atrophied (hippocampus; 6,819 mm^3^). Two clusters of atrophy were observed in the LHA group, the most significant being left hippocampus (total 5,863 mm^3^). In the normal group, six clusters were atrophied, the main one located in middle cingulate gyrus (totaling 6,5076 mm^3^). All clusters are listed in Table 3.

**Table 3:** Results of voxel-based morphometry (VBM) and correlation analysis between gray matter volumes and interictal discharges source maps (EEG) for patients with mesial temporal lobe epilepsy (bilateral, right and left hippocampal atrophy, and without hippocampal atrophy) with normal controls.

**Group**	**Method**	**p (FWE)/r**	**Size (mm)**	**T or Z**	**Coordinates**	**Anatomy**
Bilateral	VBM	0	67,203	8.57	30 −16 −18	Hippocampus
	VBM	0	7,298	5.31	52 −25 8	Superior temporal
	VBM	0	6,9073	5.22	7 −12 42	Middle cingulate
	VBM	0.015	3,518	4.86	34 −78 39	Middle occipital
	VBM	0.011	3,783	4.57	36 46 31	Middle frontal
	VBM	0.041	2,675	4.42	25 48 −13	Anterior orbital
	VBM	0.000	13,303	4.34	−23 −70 −24	Cerebellum
	EEG	0.000/−0.87	4,932	3.49	29 1 8	Putamen/insula
Right	VBM	0.001	6,819	5.37	26 −32 −2	Hippocampus
	EEG	0.020/−0.70	1,038	3.46	32 33 8	Inferior frontal/insula
Left	VBM	0.035	2,810	4.95	−29 −15 −20	Hippocampus
	VBM	0.026	3,053	4.31	−17 14 −3	Putamen
	EEG	0.001/−0.56	1,717	2.64	−28 −5 −19	Hippocampus/insula
Normal	VBM	0	7,302	5.46	−2 −24 31	Middle cingulate
	VBM	0	10,153	5.36	44 −21 10	Superior temporal
	VBM	0	32,290	5.29	−23 12 −12	Anterior insula
	VBM	0.022	3,208	4.68	−9 −94 19	Occipital pole
	VBM	0	8,153	4.63	−4 −10 16	Thalamus
	VBM	0.009	3,970	4.34	−34 8 −36	Temporal pole
	EEG	0.000/−0.86	4,335	3.98	53 −21 56	Pre/postcentral
	EEG	0.000/−0.78	5,335	3.31	18 61 6	Superior and medial frontal

No areas of increased gray matter volumes were observed.

### Correlation Analysis

**Figure 6:**
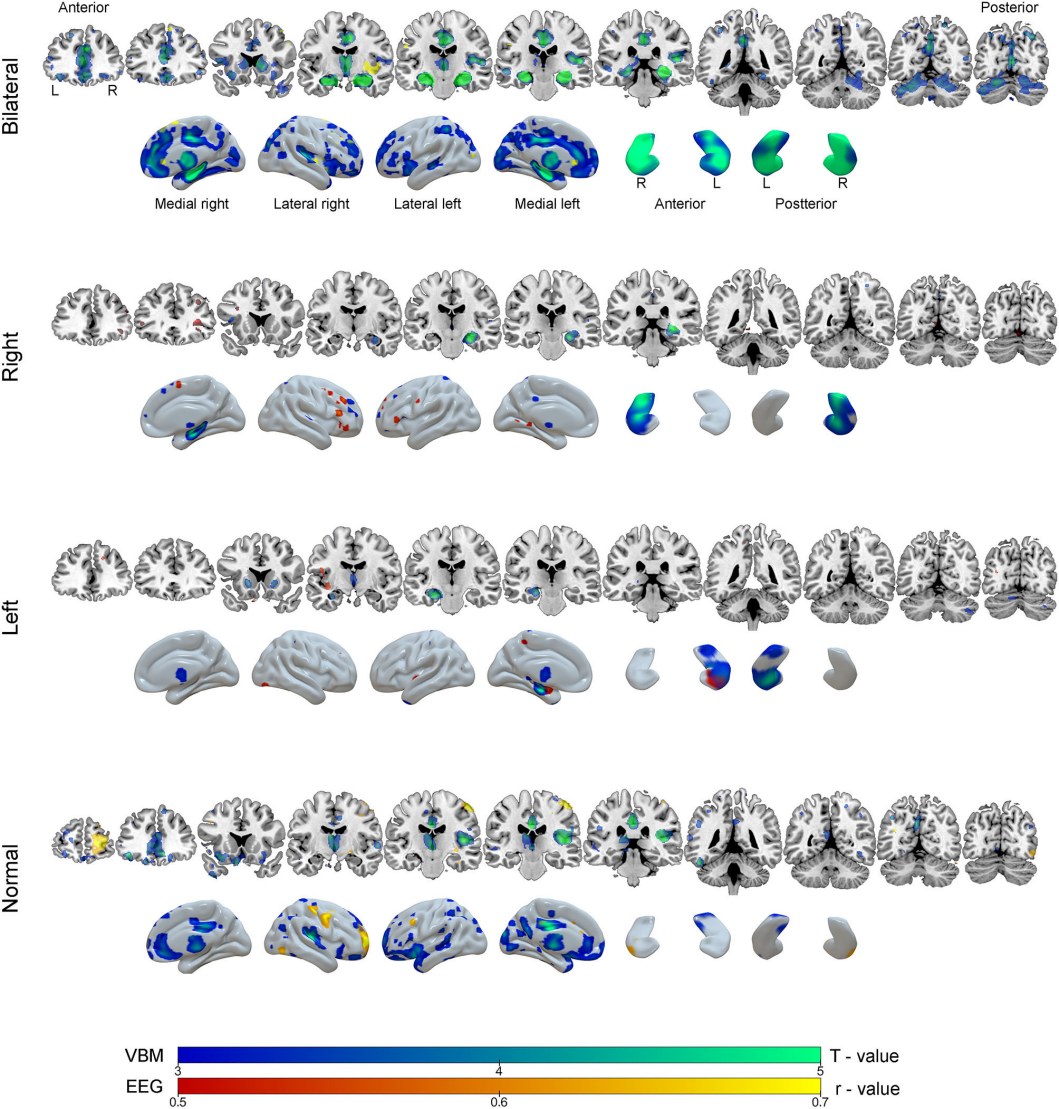
Results of the voxel-based morphometry (VBM, blue-green color scale) and correlation analysis between EEG source images and gray matter maps (red-yellow color scale) according to groups of mesial temporal lobe epilepsy. The results are overlaid on coronal MRI model slices in neurological orientation (right to right) and in three-dimensional models of the brain and hippocampus. The color scale in the figure indicates the results of statistical analysis for VBM (T - value) and correction analysis between EEG and gray matter (r - value, negative correlations).

Correlations were drawn between source maps of interictal epileptiform discharges and gray matter maps. For the BHA group, a strong negative correlation was observed in right insula (*r* = −0.87, *p* < 0.0001). In RHA group, the right inferior frontal gyrus was significant (*r* = 0.70, *p* = 0.020). In the LHA group, left hippocampus was negatively correlated (*r* = −0.56, *p* = 0.001). For the normal group two clusters of correlated regions were observed, the main one located in postcentral gyrus (*r* = −0.86, *p* < 0.0001). No areas of positive correlations were observed. Detailed results of these analyses can be seen in Table 3 and Figure 6.

## Discussion

Using quantitative EEG source imaging the present study demonstrated that at an individual level, interictal epileptiform discharges involved the claustrum of patients with MTLE. This behavior occurred in all subgroups (BHA, RHA, LHA, and normal). Voxel wise structural analysis revealed a different pattern of gray matter atrophy for each subgroup. Correlation analysis between gray matter and interictal spike source imaging revealed that there was not a perfect overlap between major structural abnormalities and source distribution.

The claustrum is a thin, irregular neural structure located on the inner surface of the insula with connections to nearly all cortical regions, and its function is thought to include synchronization of disparate perceptual, cognitive, and motor modalities (22). Bilateral abnormalities in the claustrum were associated with refractory status epilepticus, focal motor seizures, and myoclonic seizures (23). Physiological properties of the claustrum may help to explain the propagation and synchronization of abnormal epileptic activity from various cortical regions, since claustro-cortical fibers connect the claustrum with several cortical areas including the prefrontal cortex, pre- and postcentral gyri, and orbitofrontal and medial temporal cortices (24).

The claustrum is composed of GABA-ergic interneurons, damage to which could promote a state of hypersynchronization in connections with distant cortical regions. The frontal piriform cortex was found in the evaluation of IED in patients with focal epilepsy in one study. Because of their location close to the claustrum, they may be responsible for the origin and spread of epileptiform activity (25).

Several theories have been proposed to explain the impairment of consciousness that occurs during focal seizures: reduction of cerebral blood flow to areas responsible for consciousness in response to hyperflow in temporal regions; bilateral activation of hippocampus after unilateral seizure and, more recently, a loss of activity of the default mode network with bilateral deactivation of posterior cingulate (26). The involvement of insular cortex and claustrum could be related to impairment of consciousness and the frequent involvement of orofacial musculature in focal seizures (23).

Other regions identified as sources for interictal epileptiform discharges were inferior frontal region, insula, and superior temporal gyrus. Interictal spikes may be related to cognitive decline, as observed in experimental models (27). A more widespread distribution of epileptogenic discharges as observed in some patients may influence cognition via frontal and temporal lobes and limbic system that are especially involved in cognition.

One investigation of 22 patients with HA and 12 patients without HA used equivalent dipole source localization to analyze interictal epileptiform discharges obtained during pre-surgical video-EEG monitoring. This investigation showed no differences between patients with or without HA suggesting the same etiology for these discharges (6). Using similar source analysis methodology to that used here, a study with 15 patients applied the low-resolution electromagnetic tomography (LORETA) algorithm and observed that this technique can aid in detection of discharge propagation (28).

Therefore, precise determination of the epileptogenic zone is vital for surgical planning, and the possibility of noninvasive tests being used in the preoperative routine may benefit these patients. Coito et al. (29) evaluated IED routine EEG with electrical source analysis in 34 patients (20 temporal lobe epilepsy and 14 extra temporal epilepsy) before and after surgery. The efficiency were concordant with the presumed epileptic zone in 76% of the patients, showing the importance of these findings.

Regarding the morphometric evaluation, patients with BHA presented diffuse atrophy with a centrifugal pattern compared to the other groups. In the groups with unilateral HA, the evaluation by VBM demonstrated ipsilateral atrophy, as expected. When comparisons were made, we observed that the group with right HA exhibits changes related to the right lower frontal gyrus, lower right temporal and left hippocampus in the three analyzes, respectively. Such changes could be related to anterior, mesial and contralateral spread of epileptiform discharges in the group with HA on the right. On the other hand, in the group with left HA the correlation changes were restricted to the mesial structures on the left, in the three analyzes: left parahipocampal gyrus, left upper temporal gyrus and left hippocampus. Thus, the more limited pattern of correlation on the left indicates a higher current density with a greater degree of atrophy in these structures. Thus, a different neuroanatomical involvement was discovered in patients with left or right epileptiform discharges.

Moreover, for groups with lateralized HA, VBM revealed that patients with LHA had more anterior hippocampal involvement and patients with RHA had a more posterior involvement. A previous investigation described similar findings (30). Abnormalities outside the mesial temporal lobe were also observed. Patients in LHA group presented a slightly more diffuse pattern involving bilateral basal ganglia and thalamus. The literature related to MTLE laterality and atrophy is controversial. One investigation found more structural abnormalities in patients with right MTLE, especially in ipsilateral insula and contralateral thalamus (31). Another study observed a higher degree of cerebral atrophy, longer duration of disease, and worse control of seizures in patients with left HA (32).

Patients without HA showed widespread area of atrophy involving the amygdala, cingulate, temporal, and frontal cortex. This observation is in line with other studies suggesting that MRI-negative temporal lobe epilepsy is a different condition involving separate networks (33).

Negative correlations were observed between gray matter volumes and source maps; i.e., increased current densities were associated with reduced gray matter. For unilateral MTLE, differential network involvement was found in patients with left (hippocampal region and insula) or right (inferior frontal gyrus) HA. Once again these findings align with connectivity studies to point to distinct patterns for right or left HA (34). These results show how a focal pathology can influence diffuse neural networks differently depending on the affected hemisphere. Patients with bilateral HA had areas of correlation localized to the basal ganglia, insula, and mesial frontal regions. Finally, patients without any hippocampal atrophy had an extensive area of extra-temporal correlations. This suggests that the irritative zone does not always contain the epileptogenic zone. It also indicates that neuroanatomical generators and pathways of interictal discharges are heterogeneous and vary according to MTLE subtype.

Scalp EEG has a very precise temporal resolution, but limited spatial resolution for source analysis. The number of electrodes in this study was modest; currently, there are EEG studies with a greater number of electrodes and channels, and thus greater accuracy of source detection (35). However, the methodology used here is very similar to that of daily clinical practice and should be relatively easy to implement in clinical settings in the future.

Associated anatomopathological studies may help to confirm diagnosis and elucidate the relationship between HA and epileptogenic networks in MTLE (36). Future investigations using dense array EEG, with a higher number of subjects and prospectively evaluating these patients after surgical procedure should be performed in order to confirm these findings.

## Conclusions

At the individual level, interictal epileptiform discharges localized mainly to the claustrum of patients with MTLE. Quantitative analysis of MRI showed a heterogeneous and distinct pattern for MTLE subtypes. Finally, correlation analysis between quantitative EEG and structural MRI failed to demonstrate overlap with the epileptogenic zone but illustrated different networks involved in each MTLE subgroup. The observations of the present investigation suggest different pathophysiological mechanisms for interictal epileptiform discharges even among patients with unilateral HA. These findings may contribute to a better understanding of the MTLE pathophysiology, the extent of abnormalities and, in the future, to a more individualized approach for surgical therapy.

## Data Availability Statement

All datasets generated for this study are included in the article/Supplementary Material.

## Ethics Statement

The studies involving human participants were reviewed and approved by Comitê de Ética da Faculdade de Medicina da UNESP (Processo 64784917.7.0000.5411). The patients/participants provided their written informed consent to participate in this study.

## Author Contributions

EF took the lead in writing the manuscript. All authors provided critical feedback and helped shape the research, analysis and manuscript. EF and LB designed the model and the computational framework and analyzed the data.

## Conflict of Interest

The authors declare that the research was conducted in the absence of any commercial or financial relationships that could be construed as a potential conflict of interest.
