# Supplementary material for: Analysis of Interictal Epileptiform Discharges in Mesial Temporal Lobe Epilepsy Using Quantitative EEG and Neuroimaging
Source: Front Neurol. 2020 Nov 26;11:569943. doi: 10.3389/fneur.2020.569943 (PMC7726439; doi:10.3389/fneur.2020.569943)
Supplement: Supplementary file 1 [file Data_Sheet_1.PDF]

Appendix. Clinical, electroencephalographic and neuroimaging characteristics of 71 patients with mesial temporal lobe epilepsy.

Group	First	Freq/ month	Medicine	EEG	Age (years)	Sex	Vol HD	z HD	Vol HE	z HE	z IA
Right	1	1	TOP100/ CBZ 1800/ CLB 20	Bilateral	45	M	2419,0	-3,9	3560,2	3,1	-11,8
Right	8	3	FB200/ CBZ600	Right	60	F	1888,2	-3,9	3678,8	0,1	-19,6
Right	12	4	CBZ1200/ CLB 20	Right	60	M	2428,6	-5,3	3509,1	0,7	-11,3
Right	30	6	TOP 300/ CLB 40	Right	59	F	1947,1	-6,9	3353,5	1,3	-16,2
Right	11	4	OXC 900/ AV 1000	Bilateral	31	M	2395,1	-4,5	3132,7	-0,1	-8,5
Right	26	2	CBZ 600	Right	52	F	2627,6	-3,9	3522,8	1,2	-9,2
Right	1	40	CBZ1200/ AV 500/ CLB 20	Right	46	M	2628,9	-4,4	3868,1	2,4	-11,8
Right	2	0	TOP200/ CBZ400	Left	20	F	3298,1	-1,7	3560,9	-0,1	-2,9
Right	17	1	CBZ 1200/ CLN 4	Bilateral	54	F	2437,2	-4,2	3649,2	2,9	-12,3
Right	1	1	CBZ 1000	Right	56	F	2231,6	-6,5	3470,0	0,2	-13,4
Right	3	10	CBZ 1200/ CLB40	Right	24	M	2812,4	-4,0	3468,9	-0,5	-6,8
Right	10	20	CBZ 400/ CLB 10	Right	37	F	2930,4	-3,6	3633,5	0,2	-6,9
Right	7	8	AV1000/ CBZ200	Right	33	F	2430,1	-4,2	3382,1	1,5	-10,2
Right	14	5	CBZ 600/LMT300	Right	52	F	2810,7	-2,5	3592,8	2,2	-7,8
Right	12	5	CBZ1000/ CLB40	Right	46	M	3526,9	0,7	3716,3	2,1	-2,1
Right	12	4	CBZ 1600/ CLB30	Right	55	F	2552,0	-4,6	3108,2	-1,5	-6,4
Right	1	90	CBZ 1800 TOP 400 CLB 60 LMT 100	Right	36	F	2698,7	-4,3	3333,6	-0,9	-6,8
Right	16	7	LEV750/ CBZ1200/ CLB20	Right	51	F	2817,5	-2,9	3498,7	1,0	-7,0
Right	22	15	LMT300/ CLN 8	Right	39	F	2442,4	-4,7	3485,7	1,3	-11,0
Right	8	4	TOP150/ CBZ1200/ CLB5	Right	35	F	2593,8	-4,5	3430,0	0,1	-8,8
Right	39	0	CBZ800/ CLB20	Bilateral	56	F	2748,1	-1,8	3679,2	4,1	-9,1
Left	13	4	LMT400/ CLB20	Left	50	M	3187,0	-0,2	2509,7	-3,7	6,4
Left	44	1	CBZ1800/ CLB60	Left	60	F	3482,6	2,2	2347,2	-4,0	10,9
Left	16	4	CBZ600/ FB100	Right	37	F	3071,5	1,1	2201,1	-4,0	9,1
Left	2	2	TOP300/ CBZ600/ CLB 20	Left	48	F	3339,5	-1,3	2601,9	-4,7	6,7
Left	13	2	CBZ800/ CLB40/ FB 200	Left	61	F	3570,4	0,9	2428,2	-4,9	10,6
Left	55	2	CBZ 600	Left	59	M	3431,6	0,3	1961,5	-7,3	15,5
Left	12	4	LAC200 CLB60 LMT400 TOP100 GBP600	Left	30	F	3000,2	-1,4	2202,3	-5,6	8,5
Left	16	0	CLN 4/ CBZ 1000	Left	60	F	3226,6	1,1	2884,0	-0,5	2,7
Left	24	15	CBZ 1200/ TOP 250/ CLB 20	Left	35	F	3414,2	1,3	2986,4	-0,7	3,3
Left	10	3	FNT 300/ CLB 20	Left	56	M	3305,7	3,7	2567,7	-0,7	6,8
Left	52	2	TOP 250/ FNT 400/ CLB 10	Left	59	F	3393,3	0,5	3071,6	-1,0	2,3
Left	1	1	TOP 300/ CBZ 1000	Bilateral	50	F	3807,2	7,1	2389,7	-1,9	12,9
Left	40	0	TOP 50	Right	56	F	3705,6	-1,0	3409,7	-2,1	1,9
Left	3	1	CBZ 800/ CLB 10	Left	55	F	3942,0	3,0	2486,0	-4,5	12,8
Left	13	20	CBZ 600/ CLB 20	Left	30	F	3940,2	1,6	2791,9	-3,8	9,5
Left	43	36	CBZ 600/ AV 1250/ CLB 10	Left	48	M	4260,8	6,9	2630,7	-2,2	13,4
Left	12	20	LMT400/ CLB20/ FNT200/ AV1000	Left	33	M	3945,8	-0,6	2744,6	-5,7	10,0

Group according to the hippocampal atrophy side; First: Age of the first seizure in years; Freq / month: estimated number of monthly seizures; Medicine: Daily dose in milligrams of antiepileptic drugs in use at the time of acquisition magnetic resonance imaging (CBZ - Carbamazepine, LMT - Lamotrigine, LEV - Levetiracetam; LAC - Lacosamide; CLB - Clobazam, TPM - Topiramate, FNT - Phenytoin, OXC - Oxcarbazepine, AV - Valproic Acid, FB - Phenobarbital, CLN - Clonazepan, GBP - Gabapentine); EEG: qualitative result of the exam with indication of lateralization of epileptiform activity; M: male. F: female; Age: in years; Vol HD: volume of the right hippocampus (non-normalized) in cubic millimeters; z HD: z - score of the right hippocampus; Vol HE: volume of the left hippocampus (non-normalized) in cubic millimeters; z HD: z - score of the left hippocampus; z IA: z - asymmetry index score.

Appendix. Clinical, electroencephalographic and neuroimaging characteristics of 71 patients with mesial temporal lobe epilepsy.

Grupo	Primeira	Freq/ month	Medicações	EEG	Idade (anos)	Sexo	Vol HD	z HD	Vol HE	z HE	z IA
Left	10	6	CBZ 1200/ CLB 40	Left	36	F	3395,3	0,1	2498,0	-4,4	8,4
Left	10	2	LMT 50	Left	40	M	3391,0	0,6	2594,0	-3,4	7,3
Left	1	1	CBZ 1200/ TOP 250/ CLB 20	Left	43	F	3286,4	-0,3	2456,1	-4,5	7,9
Left	13	24	CBZ 1000/ CLB 10	Bilateral	36	F	3153,7	0,5	2083,8	-5,5	11,5
Left	20	20	CLB 40/ CBZ 1200/ LMT 200	Left	37	M	3971,2	2,3	3015,9	-2,3	7,5
Left	13	20	CBZ 600/ CLB 20	Left	30	F	3940,2	1,6	2791,9	-3,8	9,5
Left	43	36	CBZ 600/ AV 1250/ CLB 10	Left	48	M	4260,8	6,9	2630,7	-2,2	13,4
Left	12	20	LMT400/ CLB20/ FNT200/ AV1000	Left	33	M	3945,8	-0,6	2744,6	-5,7	10,0
Left	10	6	CBZ 1200/ CLB 40	Left	36	F	3395,3	0,1	2498,0	-4,4	8,4
Left	10	2	LMT 50	Left	40	M	3391,0	0,6	2594,0	-3,4	7,3
Bilateral	23	2	CBZ 1000/ CLB 10	Right	27	F	2532,6	-3,3	2410,1	-3,8	0,9
Bilateral	10	0	CBZ 600	Left	59	F	2537,6	-4,4	2485,4	-4,5	0,0
Bilateral	24	12	AV 1500/ CLB 30	Left	60	M	2698,4	-3,7	1858,2	-8,0	10,3
Bilateral	2	1	CBZ 1200	Bilateral	42	M	2466,6	-4,6	2044,2	-6,8	4,9
Bilateral	2	20	CBZ 800/ CLB 20/	Right	35	F	2182,8	-5,1	1943,0	-6,4	2,8
Bilateral	2	3	FB 100/ CLB 10/ FNT 300	Left	54	F	2517,8	-3,4	2048,5	-5,9	5,5
Bilateral	1	4	CBZ 800/ CLN 3	Right	24	F	1981,2	-6,5	1878,5	-7,0	1,0
Bilateral	8	1	CBZ 1000/ CLB 20	Right	37	M	2986,3	-2,9	2922,0	-2,9	0,0
Bilateral	7	3	CBZ 1200/ CLB 40	Left	35	F	2466,7	-2,2	1828,8	-6,1	8,2
Bilateral	24	2	AV 750/ CLB 20	Bilateral	61	F	2447,7	-4,9	1932,5	-7,5	6,3
Bilateral	20	1	CBZ 1200/ CLB 20	Left	42	F	1948,9	-6,9	1953,3	-6,7	-0,7
Bilateral	1	2	AV 2000/ CLB 40	Right	18	M	3503,6	-3,0	3674,7	-2,0	-2,0
Normal	25	1	CBZ 200	Left	57	F	3306,7	-0,5	3363,3	0,1	-1,1
Normal	54	1	CBZ 400	Left	60	F	3484,4	-0,1	3356,6	-0,4	0,5
Normal	23	20	CBZ 600	Left	33	F	2882,9	-1,5	2813,7	-1,6	0,1
Normal	23	3	CBZ 400	Right	44	F	3003,3	0,0	2959,6	0,1	-0,2
Normal	37	0	CBZ 400	Left	38	F	3628,0	2,4	3731,5	3,5	-1,4
Normal	36	0	FNT 200	Right	38	M	3911,8	-0,4	3634,1	-1,3	1,6
Normal	40	0	OXC 600	Left	54	F	3743,7	2,3	3636,4	2,2	0,3
Normal	15	8	CBZ 1200/ CLB 20	Right	60	F	4066,9	4,2	3948,7	4,1	0,3
Normal	10	12	CBZ 800/ FB 100	Left	58	F	3159,2	0,0	3018,5	-0,5	0,8
Normal	30	0	LMT 50	Left	62	F	2861,4	-1,1	2772,0	-1,3	0,3
Normal	31	0	CBZ 400	Right	64	M	4235,5	2,7	3976,6	1,8	1,3
Normal	32	1	CBZ 400	Right	39	F	4140,1	0,4	3998,3	0,1	0,4
Normal	15	2	CBZ 1000/ CLB 40	Right	31	M	4245,5	0,5	4039,8	0,0	0,9
Normal	75	1	LMT 100	Left	80	F	2671,5	-1,0	2767,5	0,0	-1,6
Normal	15	0	CBZ600	Right	50	M	3643,3	4,3	3515,9	4,0	0,5
Normal	42	1	CBZ 400	Left	51	F	3145,0	0,8	3003,7	0,4	0,8

Group according to the hippocampal atrophy side; First: Age of the first seizure in years; Freq / month: estimated number of monthly seizures; Medicine: Daily dose in milligrams of antiepileptic drugs in use at the time of acquisition magnetic resonance imaging (CBZ - Carbamazepine, LMT - Lamotrigine, LEV - Levetiracetam; LAC - Lacosamide; CLB - Clobazam, TPM - Topiramate, FNT - Phenytoin, OXC - Oxcarbazepine, AV - Valproic Acid, FB - Phenobarbital, CLN - Clonazepan, GBP - Gabapentine); EEG: qualitative result of the exam with indication of lateralization of epileptiform activity; M: male. F: female; Age: in years; Vol HD: volume of the right hippocampus (non-normalized) in cubic millimeters; z HD: z - score of the right hippocampus; Vol HE: volume of the left hippocampus (non-normalized) in cubic millimeters; z HD: z - score of the left hippocampus; z IA: z - asymmetry index score.
