# Supplementary material for: Analysis of Interictal Epileptiform Discharges in Mesial Temporal Lobe Epilepsy Using Quantitative EEG and Neuroimaging
Source: Front Neurol. 2020 Nov 26;11:569943. doi: 10.3389/fneur.2020.569943 (PMC7726439; doi:10.3389/fneur.2020.569943)
Supplement: Supplementary file 2 [file Data_Sheet_2.pdf]

Supplementary tables: Results of the source localization using Classical LORETA Analysis Recursively Applied (CLARA) algorithm for mesial temporal lobe epilepsy subgroups (bilateral, right and left hippocampal atrophy, and without hippocampal atrophy).

Bilateral

	Right	Left	Total
Claustrum	2	1	3
Inferior frontal	1	2	3
Lentiform	1	1	2
Insula	1	1	2
Sub-lobar	0	1	1
Culmen	0	1	1
Superior temporal	1	0	1
Thalamus	1	0	1

Normal

	Right	Left	Total
Claustrum	1	5	6
Lentiform	4	0	4
Parahippocampal	2	0	2
Inferior frontal	2	0	2
Precentral	0	2	2
Superior temporal	0	1	1
Caudate	0	1	1

Right

	Right	Left	Total
Claustrum	6	0	6
Insula	4	2	6
Inferior frontal	3	2	5
Superior temporal	3	1	4
Parahippocampal	3	0	3
Cingulate	1	2	3
Middle frontal	1	1	2
Middle temporal	0	1	1
White matter	1	0	1

Left

	Right	Left	Total
Claustrum	2	4	6
Superior temporal	1	5	6
Inferior temporal	2	3	5
Insula	1	3	4
Parahippocampal	0	3	3
Culmen	1	1	2
Cingulate	1	1	2
Caudate	1	1	2
Lentiform	0	1	1
Inferior temporal	0	1	1
